# Beruflich erworbene SARS-CoV-2-Infektionen bei medizinischem Personal in Frankfurt am Main von März bis August 2020

**DOI:** 10.1007/s00103-022-03521-2

**Published:** 2022-04-06

**Authors:** Anton Sundberg, René Gottschalk, Sabine Wicker

**Affiliations:** 1 Gesundheitsamt, Frankfurt am Main, Deutschland; 2 Institut für Medizinische Virologie, Universitätsklinikum Frankfurt am Main, Frankfurt/Main, Deutschland; 3 Betriebsärztlicher Dienst, Universitätsklinikum Frankfurt am Main, Frankfurt/Main, Deutschland

**Keywords:** COVID-19, Arbeitsbedingt erworbene Infektion, Medizinisches Personal, Epidemiologische Surveillance, Berufskrankheit, COVID-19, Occupationally acquired infection, Healthcare personnel, Surveillance, Occupational disease

## Abstract

**Hintergrund:**

Eine standardisierte Erhebung von COVID-19-Infektionen bei Gesundheitspersonal während der laufenden Pandemie war und ist nicht gegeben. Vor allem der Anteil von arbeitsbedingten Infektionen beim Gesundheitspersonal und die Frage, welche Arbeitnehmer/-innen darunter am meisten gefährdet sind, bleiben unklar.

**Ziel:**

Ziel dieser Studie war es, die gemeldeten COVID-19-Fälle beim Gesundheitspersonal in Frankfurt/Main in den ersten 6 Monaten der Pandemie zu analysieren, die Zahl der arbeitsbedingten Infektionen zu ermitteln und somit eine bessere Interpretation der durch das Robert Koch-Institut veröffentlichten Daten zu ermöglichen.

**Methoden:**

Die Daten des Gesundheitsamts Frankfurt/Main wurden für den Zeitraum vom 01.03. bis zum 31.08.2020 betrachtet und medizinisches Personal für eine Querschnittserhebung im Rahmen einer Umfrage rekrutiert. Drei Subgruppen wurden nach Ort des Infektionskontakts, am Arbeitsplatz, im Privaten und unbekannt, unterteilt und analysiert.

**Ergebnisse:**

Medizinisches Personal machte 11,8 % (319/2700) aller gemeldeten COVID-19-Fälle in Frankfurt/Main im untersuchten Zeitraum aus. In der Umfrage gaben 47,2 % der Befragten an, dass ihre Infektion am Arbeitsplatz erworben wurde. Es zeigte sich eine Assoziation von Kontakt zu COVID-19-Patient/-innen sowie der Beschäftigung auf einer internistischen Station und einer arbeitsbedingten Infektion. Ersichtlich wurde außerdem ein Zusammenhang zwischen mutmaßlichen Infektionen am Arbeitsplatz und folglich gestellten Verdachtsanzeigen auf Berufskrankheit.

**Diskussion und Fazit:**

Gesundheitsämter sind in der Lage, relevante Daten von arbeitsbedingten Transmissionen in Berufen und Arbeitsplätzen im Gesundheitswesen zu erheben, und sollten standardisierte Daten zu infiziertem Gesundheitspersonal generieren. Diese Daten sind notwendig, um gezielte Maßnahmen der Infektionsprävention zu ergreifen, die Gesundheitspersonal und ihre Patient/-innen schützen.

**Zusatzmaterial online:**

Zusätzliche Informationen sind in der Online-Version dieses Artikels (10.1007/s00103-022-03521-2) enthalten.

## Abstract (en)

**Background:**

Standardised surveillance of COVID-19 infections among healthcare personnel during the current pandemic was and is not available. In particular, the proportion of occupational infections among healthcare personnel and which workers among them are most at risk remains unclear.

**Objectives:**

The aim of this study was to analyse the reported COVID-19 cases among healthcare personnel in Frankfurt/Main during the first six months of the pandemic, to determine the number of occupational infections and thus to allow a better interpretation of the data published by the Robert Koch Institute.

**Methods:**

Data from the Frankfurt/Main Health Protection Authority was analysed for the period from 1 March to 31 August 2020, and healthcare personnel were recruited for a cross-sectional survey. Three subgroups were defined and analysed according to whether the infectious contact occurred at work, in private or in an unknown setting.

**Results:**

Healthcare personnel accounted for 11.8% (319/2700) of all reported COVID-19 cases in Frankfurt/Main during the period studied. In the survey, 47.2% of respondents reported that their infection was acquired in the workplace. There was an association of contact with COVID-19 patients as well as employment in the internal medicine ward and a reported work-related infection. Also apparent was an association between suspected occupational infections and consequently filed reports for alleged occupational disease.

**Discussion and conclusion:**

Health protection authorities are in a position to collect relevant data on work-related transmissions in healthcare occupations and workplaces and should generate standardised data on infected healthcare personnel. This data is necessary to take targeted infection control and prevention measures that protect healthcare personnel and their patients.

## Einführung

Medizinisches Personal hat ein erhöhtes Risiko, sich mit Severe Acute Respiratory Syndrome Coronavirus Type 2 (SARS-CoV-2) zu infizieren und an Coronavirus Disease 2019 (COVID-19) zu erkranken [1–4]. Das Ausmaß und die Definitionen der Meldung von COVID-19-Fällen bei Gesundheitspersonal an die Überwachungssysteme sind jedoch international sowie national uneinheitlich. Die Weltgesundheitsorganisation (WHO) und nationale Gesundheitsbehörden, unter anderem das Robert Koch-Institut (RKI), beklagen das Fehlen kohärenter Daten über die berufliche Tätigkeit von infiziertem Personal und darüber, ob eine SARS-CoV-2-Infektion am Arbeitsplatz erfolgte und damit als arbeitsbedingt angesehen werden kann [5–10].

Im September 2020, nach der ersten Welle und dem Sommerplateau, meldete das RKI insgesamt 243.599 SARS-CoV-2-Fälle [11]. Bei Personal des Gesundheitswesens wurde zwischen Personen mit Tätigkeit in medizinischen Einrichtungen und in Gemeinschaftseinrichtungen nach dem Infektionsschutzgesetz (IfSG; *n* = 15.125 und *n* = 10.613) unterschieden. Letztere umfassen Pflegeeinrichtungen, aber auch Justizvollzugsanstalten, Flüchtlingsunterkünfte und Übernachtungsmöglichkeiten für obdachlose Menschen, was eine Identifikation von medizinischem Personal erschwert [12, 13].

Zwischen Januar und Juli 2021 veröffentlichte das RKI differenzierte Informationen darüber, in welcher Art medizinischer Einrichtung und Gemeinschaftseinrichtung Personal positiv auf SARS-CoV‑2 getestet wurde [14]. In diesem Zeitraum konnten 131.834 Fälle aufgrund ihrer Kategorisierung als Gesundheitspersonal identifiziert werden, was 81,5 % (131.834/161.852) der Personen, die pauschal mit Tätigkeit in medizinischen Einrichtungen oder Gemeinschaftseinrichtungen erhoben wurden, und 3,5 % (131.834/3.741.781) aller Fälle in Deutschland zu diesem Zeitpunkt entsprach [9]. Das RKI betont dabei, dass die Daten zu infiziertem Gesundheitspersonal aufgrund fehlender Angaben zur Tätigkeit in medizinischen Einrichtungen bei gemeldeten Fällen nicht repräsentativ und als Mindestangabe zu verstehen sind [9]. Gleichzeitig fehlen diese Daten für die Frühphase der Pandemie, als persönliche Schutzausrüstung (PSA) im medizinischen Bereich knapp war. Unklar bleibt auch, erstens welche Berufe und Arbeitsplätze innerhalb des Gesundheitswesens am stärksten betroffen waren, zweitens wie viele Infektionen mutmaßlich am Arbeitsplatz erworben wurden und drittens ob die Infektionen als Verdacht einer Berufskrankheit (BK 3101) gemeldet wurden und ob diese von den zuständigen Unfallversicherungsträgern als Berufskrankheit anerkannt wurden [9].

Ziel dieser Studie war es, die Daten des Gesundheitsamts (GA) Frankfurt/Main von März bis August 2020 zu SARS-CoV-2-positivem Gesundheitspersonal zu analysieren, den Anteil der arbeitsbedingten Infektionen anhand einer Umfrage zu ermitteln und berufliche Bedingungen zu identifizieren, die mit Infektionen am Arbeitsplatz bei SARS-CoV-2-positivem medizinischen Personal in der Frühphase der Pandemie in Verbindung standen.

## Methoden

### Datenerhebung und Studiendesign

Seit dem 01.02.2020 sind Infektionen mit SARS-CoV‑2 dem zuständigen GA zu melden. Die in Frankfurt/Main erhobenen Falldaten umfassen unter anderem epidemiologische und klinische Informationen sowie eine Tätigkeit oder Unterbringung in Gesundheits- und Gemeinschaftseinrichtungen. Erfasst werden dabei Personen und somit Personal mit Wohnsitz in Frankfurt und nicht in Frankfurt Tätige, die anderswo wohnhaft sind. Ergänzt werden die Informationen durch die Fallnachbetreuung des GA. Die Daten werden über SurvNet@RKI, die bundesweite Software zur Meldung meldepflichtiger Krankheiten, an das RKI übermittelt.

In dieser Studie wurden die gemeldeten Polymerase-Kettenreaktion-(PCR-)positiven COVID-19-Fälle in Frankfurt zwischen dem 01.03. und dem 31.08.2020 analysiert.

Nach dem IfSG als in Gesundheits- (u. a. Kliniken, Praxen) oder Gemeinschaftseinrichtungen (u. a. Kindergärten, Justizvollzugsanstalten, Pflegeheime) beschäftigt eingestufte Fälle wurden identifiziert und für die weitere Analyse ausgewählt. Die durch das GA geführten Kontaktprotokolle wurden nach Informationen über genauen Beruf und Arbeitsplatz der infizierten Person durchsucht. Da sich die Studie auf Gesundheitspersonal gemäß der Definition der WHO [12] fokussiert, wurden Personen, die beispielsweise in Kindergärten und Flüchtlingsunterkünften arbeiten, ausgeschlossen. Die Auswertung der Daten erfolgte im Rahmen der Gesundheitsberichtserstattung nach dem Hessischen Gesetz über den öffentlichen Gesundheitsdienst.

### Befragung

Die gemeldeten Fälle wurden für die Teilnahme an einer anonymen Querschnittserhebung ausgewählt. Ein 69 Punkte umfassender Fragebogen wurde anhand von früheren Veröffentlichungen erarbeitet und durch das GA Frankfurt und den Betriebsärztlichen Dienst des Universitätsklinikums Frankfurt an den lokalen Kontext angepasst [12, 15–18]. Erhoben wurden Daten zur Person (z. B. Alter, Geschlecht, Beruf, Arbeitsplatz), zu den Arbeitsbedingungen vor der Diagnose (z. B. Kontakt zu COVID-19-Patient/-innen, Arbeitsbelastung, Nutzung von PSA) und zur Situation nach der Diagnose (z. B. Symptome, Langzeitfolgen). Darüber hinaus wurden Fragen zur wahrgenommenen Arbeitssicherheit und zur Durchführung von Infektionsschutzmaßnahmen gestellt. Insbesondere wurde das betroffene medizinische Personal nach dem wahrscheinlichen Infektionsort gefragt. Die Umfrage wurde durch das GA durchgeführt, umfasste eine elektronische Einverständniserklärung und wurde von der Ethikkommission des Universitätsklinikums Frankfurt genehmigt (ID 20-906).

### Statistik

Die Analyse wurde mit SPSS und BiAS durchgeführt. Die im Fragebogen erhobenen Daten wurden mit den beim GA verfügbaren Daten verglichen, um ihre Validität einzuschätzen. Auf Grundlage des angegebenen Infektionsorts wurden 3 Untergruppen unterschieden: Infektionskontakt am Arbeitsplatz, im privaten Umfeld und unbekannter Kontakt. Ein signifikanter Zusammenhang zwischen den Variablen und einer arbeitsbedingten Infektion wurde mithilfe von Kreuztabellen und der Anwendung des Chi-Quadrat- und Fishers-Exakt-Tests untersucht.

## Ergebnisse

Zunächst werden die Ergebnisse der Analyse der im GA verfügbaren Daten zu infiziertem medizinischen Personal vorgestellt. Anschließend werden die Ergebnisse der Umfrage in der gleichen Subgruppe im Vergleich zu den offiziellen Daten dargelegt.

### Meldedaten

**Figure 1:**
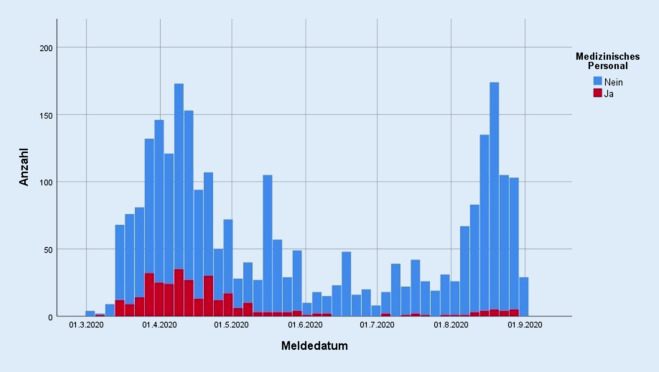

Von allen SARS-CoV‑2 per PCR positiv getesteten Einwohner/-innen der Stadt Frankfurt, die dem GA zwischen dem 01.03. und dem 31.08.2020 gemeldet wurden, waren 329 Personen als in einer Gesundheits- oder Gemeinschaftseinrichtung tätig kategorisiert. Dies entspricht 12,2 % aller Fälle zu diesem Zeitpunkt (*n* = 2700). Davon wurden 319 Personen als medizinisches Personal identifiziert (11,8 %, 319/2700; Abb. 1). 10 Personen wurden ausgeschlossen, da sie die WHO-Kriterien für die Einstufung als medizinisches Personal nicht erfüllten [12]. In 6 % der Fälle (162/2700) fehlten Angaben zur Tätigkeit in Gesundheits- oder Gemeinschaftseinrichtungen. In Deutschland wurden im gleichen Betrachtungszeitraum 10,6 % (25.738/243.599) der COVID-19-Fälle als in Gesundheits- oder Gemeinschaftseinrichtungen nach §23 bzw. §36 IfSG tätig eingestuft, was mit den 12,2 % (329/2700) in Frankfurt vergleichbar ist. Landesweit ist jedoch der Anteil des medizinischen Personals unter den in Gesundheits- und Gemeinschaftseinrichtungen Tätigen unbekannt [11].

#### Basisdaten des gemeldeten medizinischen Personals

Die 319 COVID-19-Fälle bei medizinischem Personal waren überwiegend weiblich und hauptsächlich im Alter zwischen 21 und 40 Jahren (Tab. 1).

**Table 1:** 

	GA^d^-Daten (*n* = 319)		Umfrageteilnehmende (*n* = 178)	
	*n*	%	*n*	%
**Geschlecht**				
Männlich	90	28,2	59	33,1
Weiblich	229	71,8	119	66,9
Insgesamt	319	100,0	178	100,0
**Alter in Jahren**				
Jünger als 21	14	4,4	1	0,6
21–40	171	53,6	102	57,3
41–60	118	37,0	66	37,1
61–80	16	5,0	9	5,1
Insgesamt	319	100,0	178	100,0
**Beruf**				
Arzt/Ärztin	76	32,8^a^	65	36,5
Pflegepersonal	97	41,8^a^	71	39,9
Medizinische Fachangestellte	23	9,9^a^	22	12,4
Therapeutisches Personal	5	2,2^a^	4	2,2
Andere	31	13,4^a^	38	21,3
Studierende/Auszubildende	n. v.^c^	n. v.^c^	17	9,6
Insgesamt	232	72,7	178	100,0
Nicht verfügbar	87	27,3	0	0,0
**Arbeitsplatz**				
Krankenhaus	170	55,9^a^	106	59,6
Praxis	50	16,4^a^	33	18,5
Pflegeeinrichtung	67	22,0^a^	29	16,3
Mobiler Pflegedienst	15	4,9^a^	10	5,6
Ambulante Dienste	1	0,3^a^	1	0,6
Labor	1	0,3^a^	1	0,6
Insgesamt	304	95,3	178	100,0
Nicht verfügbar	15	4,7	0	0,0
**Ausbruchsassoziation**				
Ja	170	53,3	n. v.^c^	n. v.^c^
Nein	149	46,7	n. v.^c^	n. v.^c^
Insgesamt	319	100,0	n. v.^c^	n. v.^c^
**Ort des Infektionskontakts**				
Arbeitsplatz	126	72,4^a^	84	47,2
Haushalt	31	17,8^a^	16	9,0
Andere	17	9,8^a^	79	44,4^b^
Insgesamt	174	54,5	178	100,0
Nicht verfügbar	145	45,5	0	0,0
**Symptomatisch**				
Ja	282	88,4	159	92,4^a^
Keine Symptome	37	11,6	13	7,6^a^
Insgesamt	319	100,0	172	96,6
Nicht verfügbar	0	0,0	6	3,4
**Hospitalisierung**				
Nein	302	95,0^a^	162	94,2^a^
Ja	16	5,0^a^	10	5,8^a^
Insgesamt	318	99,7	172	96,6
Nicht verfügbar	1	0,3	6	3,4
**Berufserfahrung**				
Bis zu 5 Jahre	n. v.^c^	n. v.^c^	59	33,7^a^
5–10 Jahre	n. v.^c^	n. v.^c^	40	22,9^a^
11–20 Jahre	n. v.^c^	n. v.^c^	37	21,1^a^
Mehr als 20 Jahre	n. v.^c^	n. v.^c^	39	22,3^a^
Insgesamt	n. v.^c^	n. v.^c^	174	97,8
**Durchschnittliche Dauer der Isolation**	In Tagen	95 % Konfidenzintervall (KI)	In Tagen	95 % KI
	15,40^a^	14,96–17,23	16,09^a^	14,96–17,23

^a^ berechnet auf der Grundlage gültiger Antworten

^b^ einschließlich privater Infektionen außerhalb des Haushalts und unbekannter Übertragungsorte

^c^ nicht verfügbar

^d^ *GA* Gesundheitsamt

#### Beruf und Arbeitsplatz

Angaben zu Beruf und Arbeitsplatz waren nicht Teil der standardisierten Datenerhebung des GA, die dem RKI gemeldet wurde, sondern wurden aus den Protokollen der Fallbearbeitung von SARS-CoV-2-positivem medizinischen Personal entnommen. Entsprechend fehlten Angaben zu Beruf und Arbeitsplatz in 87 (27,3 %) beziehungsweise 15 (4,7 %) Fällen. Genaue Tätigkeitsbereiche waren aus den Daten des GA nicht zu ermitteln.

Die Daten spiegeln Personal mit engem Patient/-innenkontakt wider. Die meisten gemeldeten Infektionen entfielen auf Pflegekräfte, gefolgt von Ärzt/-innen und medizinischen Fachangestellten (Tab. 1). 24 Infektionen konnten nichtmedizinischem Personal zugeordnet werden (10,3 %), sondern kamen aus den Bereichen Verwaltung (11 Personen), Hauswirtschaft (4), Gebäudemanagement (3) und Transport, Reinigung und Service (je 2). Der häufigste Arbeitsplatz war ein Krankenhaus, gefolgt von Pflegeeinrichtungen und Praxen niedergelassener Ärzt/-innen (Tab. 1).

#### Ort der Übertragung

Unter den Fällen mit Angaben zum Infektionsort (*n* = 174; 54,5 %) war die Exposition gegenüber SARS-CoV‑2 am häufigsten am Arbeitsplatz zu finden (Tab. 1). 31 (17,8 %) Kontakte fanden im Haushalt statt und 17 (9,8 %) Personen nannten andere, nicht weiter differenzierte Umstände. In 45,5 % (*n* = 145) der Fälle fehlten Angaben zum Infektionsort. Berichtete Infektionen am Arbeitsplatz waren eindeutig mit der Tätigkeit in einer stationären Einrichtung verbunden. Aus den Daten des GA konnte nicht differenziert werden, welche Abteilungen und Stationen vorrangig betroffen waren.

Bei Personen, die im Krankenhaus oder Pflegeheim arbeiteten (*n* = 132, 80,0 %), war die Wahrscheinlichkeit, den Infektionsort am Arbeitsplatz zu lokalisieren, mehr als 4,5-mal so hoch wie bei Personen, die in ambulanten Einrichtungen (*n* = 33, 20,0 %), wie z. B. Arztpraxen, arbeiteten (Odds Ratio [OR]: 4,457; 95 % Konfidenzintervall [KI]: 1,998–9,942; *p* < 0,001). Dementsprechend war die Wahrscheinlichkeit für ambulant Tätige, den Infektionskontakt am Arbeitsplatz zu verorten, geringer (OR: 0,224; 95 % KI: 0,101–0,500; *p* < 0,001), was jedoch auf einen einrichtungsbezogenen Testbias zurückzuführen sein kann. Während das GA mehr als die Hälfte aller Fälle bei medizinischem Personal als Teil eines Ausbruchs kategorisierte (*n* = 170, 53,3 %), gab es keinen signifikanten Zusammenhang mit der Lokalisierung des infektiösen Kontakts am Arbeitsplatz (*p* = 0,497), da eine Ausbruchsassoziation auch im privaten Umfeld häufig war, wie bei 18 von 31 Personen (58,1 %), die sich im eigenen Haushalt infizierten.

### Befragung

#### Rekrutierung der Umfrageteilnehmenden

Die 319 in den Daten des GA identifizierten Beschäftigten in Gesundheitseinrichtungen wurden kontaktiert und zur Teilnahme an der Umfrage eingeladen. Gemeldete, deren E‑Mail-Adressen fehlten, wurden telefonisch kontaktiert. 59 gemeldete Fälle konnten nicht erreicht werden oder lehnten die Teilnahme ab. Von den 264 Personen, die die Umfrage per E‑Mail erhalten hatten, bearbeiteten 195 den Fragebogen. 178 davon wurden als gültige Antworten gewertet, was eine Gesamtbeteiligung von 55,8 % (178/319) aller SARS-CoV-2-Fälle bei medizinischem Personal während des Zeitraums März bis August 2020 ergibt. In den 17 ausgeschlossenen, teilweise bearbeiteten Fragebögen war entweder keine Einwilligung zur Teilnahme hinterlegt oder persönliche Daten und die wahrscheinliche Infektionsquelle nicht vollständig angegeben.

#### Basisdaten

Die Umfrageteilnehmenden entsprachen in Bezug auf Geschlecht und Altersverteilung weitgehend den im GA verfügbaren Daten und repräsentierten vorwiegend junges und weibliches Personal (Tab. 1). Die 3 Subgruppen, Infektionskontakt am Arbeitsplatz, im privaten Umfeld und unbekannter Kontakt, zeigten eine gleichmäßige Verteilung der Berufserfahrung des Personals (*p* = 0,384).

#### Beruf und Arbeitsplatz

Während Informationen zum Arbeitsplatz aus Daten des GA unvollständig waren, gaben in der Umfrage alle Teilnehmenden das genaue Arbeitsumfeld an, was weitere Einblicke zu betroffenem Personal ermöglicht. Trotz der fehlenden GA-Daten zeigte sich eine ähnliche Verteilung der Berufsgruppen wie in der Umfrage (Tab. 1). Die meisten Teilnehmenden waren in einem Krankenhaus beschäftigt, gefolgt von Praxen und Pflegeeinrichtungen. Der Arbeitsplatz wurde mit einem *p*-Wert von < 0,001 mit der wahrgenommenen Infektionsquelle in Verbindung gebracht (Tab. 2). Von allen Arbeitnehmer/-innen, die in der Umfrage eine arbeitsbedingte Infektion angegeben hatten (*n* = 84), waren 75,0 % (*n* = 63) in einem Krankenhaus beschäftigt.

**Table 2:** 

	Ort des Infektionskontakts								
	Arbeitsplatz (*n* = 84)		Privat (*n* = 32)		Unbekannt (*n* = 62)		Insgesamt (*n* = 178)		*p*-Wert
	*n*	%	*n*	%	*n*	%	*n*	%	
**Geschlecht**									0,798
Männlich	29	34,5	9	28,1	21	33,9	59	33,1	0,798
Weiblich	55	65,5	23	71,9	41	66,1	119	66,9	0,798
Insgesamt	84	100,0	32	100,0	62	100,0	178	100,0	–
**Alter in Jahren**									0,294
Jünger als 21	1	1,2	0	0,0	0	0,0	1	0,6	0,570
21–40	48	57,2	24	75,1	30	48,4	102	57,3	0,047
41–60	31	36,9	7	21,9	28	45,2	66	37,1	0,086
61–80	4	4,8	1	3,1	4	6,5	9	5,1	0,773
Insgesamt	84	100,0	32	100,0	62	100,0	178	100,0	–
**Beruf**^**b**^									0,635
Arzt/Ärztin	28	33,3	15	46,9	22	35,5	65	36,5	0,391
Pflegepersonal	37	44,0	13	40,6	21	33,9	71	39,9	0,461
Medizinische Fachangestellte	9	10,7	3	9,4	10	16,1	22	12,4	0,526
Therapeutisches Personal	2	2,4	1	3,1	1	1,6	4	2,2	0,890
Nichtmedizinisches Personal	8	9,5	1	6,3	7	11,3	16	9,0	0,412
Studierende/Auszubildende	12	14,3	2	6,3	3	4,8	17	9,6	0,124
Insgesamt	84	100,0	32	100,0	62	100,0	178	100,0	–
**Arbeitsbereich**^**b**^									0,003
Innere Medizin	37	44,0	4	12,5	16	25,8	57	32,0	0,002
Chirurgie	13	15,5	4	12,5	8	12,9	25	14,0	0,873
Geriatrie/Altenpflege	12	14,3	2	6,3	9	14,5	23	12,9	0,462
Allgemeinmedizin	7	8,3	4	12,5	9	14,5	20	11,2	0,489
Zahnmedizin	2	2,4	4	12,5	3	4,8	9	5,1	0,084
Augenheilkunde	3	3,6	1	3,1	3	4,8	7	3,9	0,896
Notfallmedizin	4	4,8	0	0,0	2	3,2	6	3,4	0,445
Pädiatrie	3	3,6	2	6,3	1	1,6	6	3,4	0,493
Öffentlicher Gesundheitsdienst	2	2,4	2	6,3	2	3,2	6	3,4	0,585
Anästhesiologie	3	3,6	0	0,0	2	3,2	5	2,8	0,565
Psychiatrie	0	0,0	3	9,4	1	1,6	4	2,2	0,009
Urologie	2	2,4	2	6,3	0	0,0	4	2,2	0,152
Andere	7	8,3	5	15,6	16	25,8	28	15,7	0,016
Insgesamt	84	100,0	32	100,0	62	100,0	178	100,0	–
**Einrichtung**^**b**^									< 0,001
*Krankenhaus*	63	75,0	14	43,8	29	46,8	106	59,6	0,754
Station innere Medizin	38	61,3^a^	3	21,4^a^	9	31,0^a^	50	47,6^a^	0,004^a^
Intensivstation	18	29,0^a^	4	28,6^a^	5	17,2^a^	27	25,7^a^	0,491^a^
Ambulanz	13	21,0^a^	4	28,6^a^	8	27,6^a^	25	23,8^a^	0,686^a^
COVID-19-Station	16	25,8^a^	2	14,3^a^	5	17,2^a^	23	21,9^a^	0,522^a^
Notaufnahme	12	19,4^a^	3	21,4^a^	7	24,1^a^	22	21,0^a^	0,853^a^
Station Chirurgie	10	16,1^a^	3	21,4^a^	6	20,7^a^	19	18,1^a^	0,595^a^
Andere Stationen	12	19,4^a^	4	28,6^a^	5	17,2^a^	21	20,0^a^	0,664^a^
Operationssaal	8	12,9^a^	3	21,4^a^	7	24,1^a^	18	17,1^a^	0,355^a^
Andere Krankenhausbereiche	10	16,1^a^	3	21,4^a^	7	24,1^a^	20	19,0^a^	0,644^a^
*Praxis*	5	6,0	11	34,4	17	27,4	33	18,5	0,725
Hausärztliche Praxis	2	40,0^a^	4	36,4^a^	6	37,5^a^	12	37,5^a^	0,982^a^
Fachärztliche Praxis	0	0,0^a^	4	36,4^a^	7	43,8^a^	11	34,4^a^	0,221^a^
Zahnmedizinische Praxis	2	40,0^a^	4	36,4^a^	3	18,8^a^	9	28,1^a^	0,436^a^
Andere	1	20,0^a^	1	9,1^a^	2	12,5^a^	4	12,5^a^	0,824^a^
*Gesundheitsdienste*	17	20,2	8	25,0	19	30,6	44	24,7	0,906
Pflegeeinrichtung	12	70,6^a^	6	75,0^a^	11	61,1^a^	29	67,4^a^	0,606^a^
Mobiler Pflegedienst	3	17,6^a^	2	25,0^a^	5	27,8^a^	10	23,3^a^	0,814^a^
Andere	2	11,8^a^	2	25,0^a^	2	11,1^a^	6	14,0^a^	0,582^a^
*Insgesamt*	84	100,0	32	100,0	62	100,0	178	100,0	–
**Berufserfahrung**									0,384
Bis zu 5 Jahre	31	37,3^a^	9	28,1	19	31,7^a^	59	33,7^a^	0,591
5–10 Jahre	16	19,3^a^	12	37,5	12	20,0^a^	40	22,9^a^	0,092
10–20 Jahre	19	22,9^a^	6	18,8	12	20,0^a^	37	21,1^a^	0,857
Mehr als 20 Jahre	17	20,5^a^	5	15,6	17	28,3^a^	39	22,3^a^	0,326
Insgesamt	83	98,8	32	100,0	60	96,8	175	98,3	–

^a^ berechnet auf Basis gültiger Antworten

^b^ Mehrfachangaben möglich

Im Vergleich dazu arbeiteten lediglich 43,8 % (*n* = 14) des Personals, das ihre Infektion im Privaten lokalisierte, und 46,8 % (*n* = 29) der Beschäftigten mit unbekanntem Übertragungsort in einem Krankenhaus. Krankenhausmitarbeitende stuften ihre Infektion also eher als arbeitsbedingt (OR: 3,558; 95 % KI: 1,878–6,742; *p* < 0,001) und nicht als privat (OR: 0,457; 95 % KI: 0,210–0,991; *p* = 0,049) oder unbekannt (OR: 0,445; 95 % KI: 0,237–0,836; *p* = 0,016) ein.

Bezüglich der Berufe fehlten in den Daten des GA in knapp einem Drittel der Fälle Informationen, während alle Umfrageteilnehmenden ihren Beruf angegeben haben (Tab. 1). Wie beim Arbeitsplatz wichen die in der Umfrage gegebenen Antworten weitestgehend nicht von den Daten des GA ab. Pflegekräfte waren am häufigsten vertreten, gefolgt von Ärzt/-innen und medizinischen Fachangestellten. 16 Arbeiter/-innen (9,0 %) hatten einen nichtmedizinischen Hintergrund, wovon der Großteil in einer Pflegeeinrichtung beschäftigt war (*n* = 8; *p* < 0,001). Es gab keinen Zusammenhang zwischen einem nichtmedizinischen Beruf und dem angegebenen Ort der Infektion (*p* = 0,412; Tab. 2). 9,6 % (*n* = 17) waren Studierende oder Auszubildende, was in den offiziellen Daten des GA nicht erfasst wurde.

#### Abteilungen und Stationen

Im Gegensatz zum GA wurde in der Umfrage die Abteilung erfragt, in der das medizinische Personal hauptsächlich tätig war (Tab. 2). Der Arbeitsbereich war signifikant mit der Infektionsquelle verbunden (*p* = 0,003). In der inneren Medizin tätige Personen gaben fast dreimal häufiger an, dass ihre Infektion auf eine Exposition am Arbeitsplatz zurückzuführen war als auf ein privates oder unbekanntes Umfeld (OR: 2,913; 95 % KI: 1,512–5,610; *p* < 0,001). So war fast die Hälfte der Arbeitnehmer/-innen mit arbeitsbedingt erworbenen Infektionen in der inneren Medizin tätig (Tab. 2).

Passend dazu war die Arbeit auf einer internistischen Krankenhausstation mit einer erhöhten Wahrscheinlichkeit, die Infektion am Arbeitsplatz (OR: 3,927; 95 % KI: 1,702–9,059; *p* = 0,001), und einer geringeren Wahrscheinlichkeit, die Infektion im privaten Umfeld (OR: 0,261; 95 % KI: 0,068–0,998; *p* = 0,047) zu lokalisieren, verbunden. Abgesehen davon war die Arbeit auf bestimmten Stationen, in Praxen oder anderen spezifischen Einrichtungen nicht mit einer Infektion am Arbeitsplatz in unserer Kohorte verbunden (Tab. 2). Die Angabe einer arbeitsbedingten Infektion war nicht signifikant mit einer Tätigkeit auf mutmaßlichen Hochrisikostationen wie der COVID-19-Station, der Intensivstation oder der Notaufnahme assoziiert (Tab. 2).

#### Ort der Infektion

Die Umfrageteilnehmenden verorteten ihre Infektion mit SARS-CoV‑2 am häufigsten am Arbeitsplatz (47,2 %, 84/178; Tab. 3). Die meistgenannte mutmaßliche Infektionsquelle waren Patient/-innen, die im Nachhinein positiv auf SARS-CoV‑2 getestet wurden (41,7 %, 35/84). Es folgten der Kontakt zu infektiösen Kolleg/-innen, zu bekanntermaßen positiven Patient/-innen und der Kontakt zu einem symptomatischen Patienten ohne Laborbestätigung (Tab. 2). In 32 Fällen wurde der Infektionskontakt im privaten Umfeld lokalisiert (18,0 %, 32/178), die Hälfte davon gaben dabei den eigenen Haushalt (50,0 %, 16/32) und 17 (53,5 %, 17/32) andere private Umgebungen an. Im Vergleich zu den 17,8 % der Fälle in den Daten des GA, lokalisierten in der Umfrage folgend 9,0 % des infizierten medizinischen Personals den Infektionskontakt im eigenen Haushalt (Tab. 1).

**Table 3:** 

	Ort des Infektionskontakts								
	Arbeitsplatz (*n* = 84)		Privat (*n* = 32)		Unbekannt (*n* = 62)		Insgesamt (*n* = 178)		*p*-Wert
	*n*	%	*n*	%	*n*	%	*n*	%	
**Kontakt zu COVID-19-Patient/-innen**									< 0,001
*Ja*	74	89,2^a^	12	38,7^a^	22	35,5	108	61,4^a^	< 0,001
PCR-positive Patient/-innen	36	43,4^a^	9	29,0^a^	10	16,1	55	31,3^a^	0,002
Im Nachhinein PCR-positive Patient/-innen	50	60,2^a^	3	9,7^a^	10	16,1	63	35,8^a^	< 0,001
Symptomatische Patient/-innen mit unbekanntem PCR-Status	34	41,0^a^	3	9,7^a^	9	14,5	46	26,1^a^	< 0,001
*Nein/Unbekannt*	9	10,8^a^	19	61,3^a^	40	64,5	68	38,6^a^	< 0,001
Nein	6	7,2^a^	16	51,6^a^	25	40,3	47	26,7^a^	< 0,001
Unbekannt	5	6,0^a^	3	9,7^a^	16	25,8	24	13,6^a^	0,002
*Insgesamt*	83	98,8	31	96,9	62	100,0	176	98,9	–
**Patient/-innenkontakt von unter 2** **m Entfernung**									0,055
Mit PSA^b^	57	73,1^a^	13	86,7^a^	29	76,3^a^	99	75,6^a^	0,529
Ohne PSA^b^	31	39,7^a^	0	0,0^a^	11	28,9^a^	42	32,1^a^	0,009
Nein	3	3,8^a^	2	13,3^a^	2	5,3^a^	7	5,3^a^	0,326
Insgesamt	78	92,2	15	46,9	38	61,3	131	73,6	–
**Risikostratifizierung am Arbeitsplatz**^**c**^									0,674
Hoch	29	36,7^a^	5	33,3^a^	9	23,1^a^	43	32,3^a^	0,329
Mittel	46	58,2^a^	9	60,0^a^	27	69,2^a^	82	61,7^a^	0,507
Niedrig	4	5,1^a^	1	6,7^a^	3	7,7^a^	9	6,0^a^	0,847
Insgesamt	79	94,0	15	46,9	39	62,9	133	74,7	–
**Wahrscheinliche Infektionsquelle**									< 0,001
PCR-positive/r Patient/-in	24	28,6	0	0,0	0	0,0	24	13,5	< 0,001
Im Nachhinein PCR-positive/r Patient/-in	35	41,7	0	0,0	0	0,0	35	19,7	< 0,001
Wahrscheinliche/r COVID-19-Patient/-in	20	23,8	0	0,0	0	0,0	20	11,2	< 0,001
PCR-positive/r Kolleg/-in	28	33,3	0	0,0	0	0,0	28	15,7	< 0,001
Haushalt	0	0,0	16	50,0	0	0,0	16	9,0	< 0,001
Privates Umfeld	0	0,0	17	53,1	0	0,0	17	9,6	< 0,001
Unbekannt	0	0,0	0	0,0	62	100,0	62	34,8	< 0,001
Insgesamt	84	100,0	32	100,0	62	100,0	178	100,0	–
**Verdachtsmeldung als Berufskrankheit**									< 0,001
*Ja*	42	50,0	7	21,9	12	19,7^a^	61	34,5^a^	< 0,001
Durch betriebsärztlichen Dienst	18	21,4	1	3,1	4	6,6^a^	23	13,0^a^	0,006
Durch Hausarzt/-ärztin	2	2,4	3	9,4	1	1,6^a^	6	3,4^a^	0,115
Durch die Arbeitsstelle	15	17,9	1	3,1	6	9,8^a^	22	12,4^a^	0,074
Selbstständig	7	8,3	2	6,3	1	1,6^a^	10	5,6^a^	0,223
*Nein*	42	50,0	25	78,1	49	80,3^a^	116	65,5^a^	< 0,001
Keine Meldung	17	20,2	19	59,4	37	60,7^a^	73	41,2^a^	< 0,001
Weiß nicht	25	29,8	6	18,8	12	19,7^a^	43	24,3^a^	0,271
*Insgesamt*	84	100,0	32	100,0	61	98,4	177	99,4	–

^a^ berechnet auf Basis gültiger Antworten

^b^ PSA = persönliche Schutzausrüstung

^c^ hoch = Kontakt zu Patient/-innen unter 2 m Entfernung *und* Anwesenheit bei aerosolbildenden Maßnahmen, mittel = Kontakt zu Patient/-innen unter 2 m Entfernung *oder* Anwesenheit bei aerosolbildenden Maßnahmen, niedrig = *weder* Kontakt zu Patient/-innen unter 2 m Entfernung *noch* Anwesenheit bei aerosolbildenden Maßnahmen

Den übrigen 62 Personen (34,8 %, 62/178) war unbekannt, wo der Infektionskontakt stattfand. Der Großteil (64,5 %, 40/62) davon hatte keinen Kontakt zu COVID-19-Patienten am Arbeitsplatz (OR: 14,268; 95 % KI: 6,344–32,088; *p* < 0,001; Tab. 3). Die Ergebnisse unterscheiden sich von den im GA verfügbaren Informationen, wo erstens bei 45,5 % (*n* = 145) der Fälle Angaben zum Ort der COVID-19-Exposition fehlten und zweitens der Anteil der vermuteten Infektionen am Arbeitsplatz bei den Fällen im GA höher war als bei den Umfragedaten (72,4 % gegenüber 47,2 %; Tab. 1).

#### Arbeitsbedingte Infektion

Neben den bereits erläuterten Charakteristika von betroffenem Personal sind weitere in der Umfrage erhobene Daten signifikant mit einem Infektionskontakt am Arbeitsplatz assoziiert. Dazu gehören die Wahrnehmung, dass der Arbeitsschutz unzureichend ist (*p* < 0,001), ein wahrgenommener Mangel an bereitgestellten Informationen über COVID-19 sowie über die Übertragungswege (*p* = 0,031; siehe Onlinematerial, Tab. Z1) und die Listung als Kontaktperson durch das GA vor der eigenen Diagnose (*p* = 0,026; siehe Onlinematerial, Tab. Z2).

Ein wichtiger Faktor, der mit einer arbeitsbedingten Infektion assoziiert war, war der Kontakt zu PCR-positiven Patient/-innen (*p* < 0,001; Tab. 3). Personal, das eine arbeitsbedingte Infektion angab, hatte mit dreimal höherer Wahrscheinlichkeit Kontakt zu bestätigten COVID-19-Patient/-innen (OR: 2,983; 95 % KI: 1,534–5,802; *p* = 0,001) und mit mehr als viermal höherer Wahrscheinlichkeit Kontakt zu aufgrund der Symptomatik mutmaßlichen COVID-19-Fällen (OR: 4,684; 95 % KI: 2,218–9,892; *p* < 0,001). Die Wahrscheinlichkeit, Kontakt mit einem Patienten zu haben, der kurz darauf positiv getestet wurde, war noch höher für infiziertes Personal, das eine arbeitsbedingte Infektion angab (OR: 9,324; 95 % KI: 4,482–19,399; *p* < 0,001). Dementsprechend machte Gesundheitspersonal, das keinen wissentlichen Kontakt zu COVID-19-Patient/-innen hatte, nur 10,8 % (*n* = 9) der Fälle in der Subgruppe der am Arbeitsplatz Infizierten aus, verglichen mit 61,3 % (*n* = 19) bei den im Privaten Infizierten und 64,5 % (*n* = 40) in der Subgruppe mit unbekannter Lokalisation des Infektionskontakts. Dies deutet darauf hin, dass eine Übertragung am Arbeitsplatz unabhängig von Patient/-innen, wenngleich seltener, gegeben war.

Gleichzeitig war ein arbeitsbedingter Infektionskontakt nicht signifikant mit enger Tätigkeit an Patient/-innen von weniger als 1,5 m Entfernung verbunden (*p* = 0,055; Tab. 3). Es bestand jedoch ein Zusammenhang mit Personal, das ohne adäquate PSA eng an Patient/-innen arbeitete (*p* = 0,009). Eine Risikostratifizierung von Beschäftigten ergab, dass enger Patient/-innenkontakt und/oder die Anwesenheit bei aerosolbildenden Maßnahmen, nicht mit der Lokalisation der Infektionsquelle verbunden war (*p* = 0,674).

In 61 Fällen des positiv getesteten Personals (34,5 %) wurde eine Verdachtsanzeige auf BK 3101 an den zuständigen Unfallversicherungsträger gestellt (Tab. 3). In der Befragung gaben davon 42 Personen eine arbeitsbedingte Infektion an, was einen signifikanten Zusammenhang mit der Einreichung einer offiziellen Meldung zeigt (OR: 3,895; 95 % KI: 2,011–7,544; *p* < 0,001). Folglich wurde nur bei 50,0 % (*n* = 42) des medizinischen Personals, das eine Infektion am Arbeitsplatz vermutete, der Verdacht auf BK 3101 gemeldet. Somit wurde für 42 Arbeitnehmer/-innen, die eine arbeitsbedingte Infektionsquelle angaben, kein Antrag auf Anerkennung einer BK eingereicht. Andererseits wurde in 19 Fällen eine Verdachtsanzeige gestellt, wobei die Betroffenen den Infektionskontakt nicht am Arbeitsplatz lokalisierten.

## Diskussion und Fazit

Während das erhöhte Risiko einer Infektion mit COVID-19 für medizinisches Personal im Vergleich zur Allgemeinbevölkerung bekannt ist, können die vorliegenden Ergebnisse zu einem besseren Verständnis der offiziellen Meldedaten über SARS-CoV-2-positive Personen mit Tätigkeit in Gesundheitseinrichtungen beitragen. Dies ist besonders wertvoll, da die vielen Studien unabhängig von GÄ durchgeführt wurden und sich auf bestimmte Krankenhäuser oder andere Gesundheitseinrichtungen konzentrierten [1, 2, 8, 19–24].

In unserer Grundgesamtheit, die alle in Frankfurt/Main während der ersten 6 Monate der Pandemie gemeldeten Fälle umfasst, haben wir gezeigt, dass zwar nicht alle COVID-19-Fälle unter Beschäftigten im Gesundheitswesen arbeitsbedingt sind, jedoch fast die Hälfte (47,2 %, 84/178) den Arbeitsplatz als Infektionsquelle nennt. Die Erkenntnisse unterstreichen die von der US-amerikanischen zentralen Gesundheitsbehörde Centers for Disease Control and Prevention (CDC) im April 2020 veröffentlichten Daten, in denen medizinisches Personal in 55 % der Fälle eine arbeitsbedingte Exposition angab [7]. Während die CDC-Daten nach möglichen Expositionsumgebungen fragten, wurde in unserer Studie nach der mutmaßlichen Quelle des infektiösen Kontakts gefragt. Außerdem fehlte für 84 % der Fälle die Information zur beruflichen Tätigkeit im Gesundheitswesen, während es in unseren Daten nur 6 % waren.

Im Gegensatz dazu fanden Jin et al. eine wahrgenommene arbeitsbedingte Übertragung bei 84,9 % von 105 Fällen bei Gesundheitspersonal, die sich vor dem 30.01.2020 in einem Krankenhaus der Universität Wuhan, China, ereigneten [17]. Dies ist vermutlich auf den frühen Zeitpunkt der Studie zurückzuführen, da sowohl die PSA als auch das Wissen über die Übertragung knapp waren und begrenzte Testkapazitäten wahrscheinlich zu weniger Tests in der Bevölkerung und damit zu einer Unterschätzung der Transmission in der Allgemeinbevölkerung führten.

Darüber hinaus ermöglicht unsere Studie eine Quantifizierung des Anteils medizinischen Personals unter denjenigen Fällen, die durch das RKI nach dem IfSG als in Gesundheits- oder Gemeinschaftseinrichtungen tätig gemeldet wurden. Fast alle Fälle (97,0 %), die mit Tätigkeit nach §23 und §36 IfSG eingestuft wurden, können für die erste Welle und das darauffolgende Sommerplateau in Frankfurt als medizinisches Personal kategorisiert werden. Diese Erkenntnis wurde in Deutschland bisher noch nicht untersucht und stellt eine Bereicherung für die Interpretation der Überwachungsdaten dar. Sie untermauert zudem die Einschätzung, dass etwa einer von 10 Fällen während der ersten Monate der Pandemie bei Beschäftigten im Gesundheitswesen auftrat [5].

Ziel der Studie war es zudem, Faktoren zu ermitteln, die mit einer arbeitsbedingten, PCR-bestätigten SARS-CoV-2-Infektion in Verbindung stehen. Die Studienpopulation spiegelte weitgehend Gesundheitspersonal wider, von dem bekannt ist, dass es einem größeren Risiko für eine Infektion mit COVID-19 ausgesetzt ist [1, 8, 20, 21, 25]. Hervorzuheben ist, dass die Zahl des infizierten medizinischen Personals auch in der Frühphase der Pandemie nicht mit der Zahl der arbeitsbedingten Infektionen im Gesundheitswesen gleichzusetzen ist, da Übertragungen im Privatleben weitverbreitet sind [22–24].

Bei der Betrachtung von Merkmalen des Personals, das eine arbeitsbedingte Infektion angab, fanden wir eine signifikante Assoziation des Kontakts zu COVID-19-Patient/-innen mit der eigenen Infektion bei Beschäftigten, die in Krankenhäusern arbeiteten, insbesondere auf internistischen Stationen. Berufliche Tätigkeiten und vermeintliche Hochrisikoarbeitsplätze wie die COVID-19-Station, die Notaufnahme oder die Intensivstation waren nicht mit einer vermehrten Angabe von arbeitsbedingten Übertragungen verbunden, was auf das konsequente Befolgen von Infektionsschutzmaßnahmen zurückzuführen sein könnte.

Eine Infektion am Arbeitsplatz wurde oft mit Kontakt zu Patient/-innen in Verbindung gebracht, die zu einem späteren Zeitpunkt positiv auf SARS-CoV‑2 getestet wurden. Dies deutet darauf hin, dass die Arbeit mit Patient/-innen, bei denen eine COVID-19-Infektion nicht bestätigt bzw. vermutet ist, ein Risiko für eine arbeitsbedingte Übertragung darstellt. So lässt sich annehmen, dass unerkannte COVID-19-Patient/-innen vor allem auf internistischen Stationen behandelt werden, wobei sie eine Ansteckungsgefahr für Personal darstellen. Gleichzeitig könnte ein geringeres wahrgenommenes Infektionsrisiko zu inkonsequentem Infektionsschutz auf Nicht-COVID-19-Stationen beitragen. In Abb. 1 ist beispielsweise ersichtlich, dass der Anteil des medizinischen Personals an den Gesamtinfektionen in Frankfurt im Sommer 2020 geringer ist im Vergleich zum Beginn der Pandemie. Dies bedeutet, dass eine Verringerung nosokomialer Infektionen durch umfassende Tests, die Verfügbarkeit und den stringenten Einsatz angemessener PSA sowie andere Maßnahmen des Infektionsschutzes auch in der Frühphase einer Pandemie ein erreichbares Ziel ist. Dies gilt speziell auch in Bereichen wie internistischen Stationen, die im Laufe der Pandemie nicht explizit auf die Behandlung von COVID-19 fokussiert sind.

Weiter konnte eine Diskrepanz zwischen der Angabe einer arbeitsbedingten Infektion bei betroffenem medizinischem Personal und einer gestellten Verdachtsanzeige von COVID-19 als BK 3101 festgestellt werden. Während einige der Verdachtsanzeigen bei Personal erfolgte, das die Übertragung selbst nicht am Arbeitsplatz lokalisierte, wurde bei der Hälfte jener, die eine arbeitsbedingte Infektion angaben, keine Meldung als BK 3101 veranlasst. Hier könnte eine standardisierte Datenerhebung für medizinisches Personal zu beruflichen Informationen, dem Übertragungsweg und der Assoziation mit Ausbruchsgeschehen durch das GA zu einer genaueren Identifizierung jener führen, bei denen COVID-19 als Verdachtsanzeige einer BK 3101 zu melden ist.

Die Studie hat jedoch mehrere Limitationen. Sie bezieht sich auf die erste pandemische Welle und damit auf das Wildtypvirus und im Vergleich niedrige Inzidenzen, sodass eine undifferenzierte Übertragbarkeit auf die aktuelle Verbreitungsdynamik nicht vorgenommen werden kann. Wie die meisten epidemiologischen Daten während der Pandemie basiert sie zudem auf Selbstauskünften, die Erinnerungsverzerrungen (Recall Bias) unterliegen. Auch der fehlende Zugang zu einem E‑Mail-Konto stellt eine Einschränkung für die digitale Umfrage dar und resultiert in einem möglichen Ausschluss von älteren und/oder sozial ausgegrenzten Personen, von Personen mit geringem Einkommen und von Personal, deren primäre Umgangssprache nicht Deutsch ist. Diese Einschränkungen müssen künftig berücksichtigt werden, um zu verhindern, dass diese Gruppen bei Datenerhebungen weiterhin ausgeschlossen werden.

## Fazit

Eine standardisierte Erhebung und damit Identifikation der von erhöhtem Infektionsrisiko betroffenen Arbeitsbereiche und -gruppen ist grundlegend für zielgerichtete Untersuchungen bei gefährdetem Personal im Gesundheitswesen. So könnten künftig genau die risikobehafteten Tätigkeiten, Situationen und Räumlichkeiten identifiziert und Infektionen folglich vorgebeugt werden.

Zuletzt schließen wir uns der Forderung nach einer standardisierten Datenerhebung bei medizinischem Personal, einschließlich des Berufs, des Arbeitsplatzes, der Exposition gegenüber COVID-19-Patienten und des vermuteten Übertragungswegs, durch Gesundheitsbehörden an [10]. Wir sind der Ansicht, dass GÄ entscheidend dazu beitragen können, die Lücke bei der Datenerhebung zu Infektionen bei medizinischem Personal zu schließen. Die Daten werden benötigt, um besser zu verstehen, welches medizinische Personal von arbeitsbedingten COVID-19-Infektionen bedroht ist und so eine gezielte Prävention zu ermöglichen.

## Supplementary Information




