# Supplementary material for: Occupationally acquired SARS-CoV-2 infections among healthcare personnel in Frankfurt am Main from March to August 2020
Source: Bundesgesundheitsblatt Gesundheitsforschung Gesundheitsschutz. 2022 Apr 6;65(6):639–49. [Article in German] doi: 10.1007/s00103-022-03521-2 (PMC8984668; doi:10.1007/s00103-022-03521-2)
Supplement: Supplementary file 1 [file 103_2022_3521_MOESM1_ESM.pdf]

Bundesgesundheitsblatt – Gesundheitsforschung – Gesundheitsschutz, Ausgabe 6/2022

Onlinematerial zum Beitrag:

Beruflich erworbene SARS-CoV-2-Infektionen bei medizinischem Personal in Frankfurt am Main von März bis August 2020

Anton Sundberg[1,3], René Gottschalk[1,2], Sabine Wicker[3]

[1] Gesundheitsamt, Frankfurt am Main, Deutschland
[2] Institut für Medizinische Virologie, Universitätsklinikum Frankfurt am Main, Frankfurt/Main, Deutschland
[3] Betriebsärztlicher Dienst, Universitätsklinikum Frankfurt am Main, Frankfurt/Main, Deutschland

Korrespondenzadresse

Anton Sundberg
Universitätsklinikum Frankfurt am Main
Theodor-Stern-Kai 7
60590 Frankfurt/Main
Deutschland
anton.sundberg@stud.uni-frankfurt.de

Inhalt:

Tabelle Z1: Arbeitsbedingungen des an der Querschnittsbefragung teilnehmenden medizinischen Personals, Frankfurt/Main, März - August 2020 (n = 178)
Tabelle Z2: SARS-CoV-2-Infektion und Diagnose bei dem an der Querschnittserhebung teilnehmenden medizinischen Personal, Frankfurt/Main, März - August 2020 (n = 178)

Zusatztabelle Z1: Arbeitsbedingungen des an der Querschnittsbefragung teilnehmenden medizinischen Personals, Frankfurt/Main, März - August 2020 (n = 178)

	Ort des Infektionskontakts								
	**Arbeitsplatz (n = 84)**		**Privat (n = 32)**		**Unbekannt (n = 62)**		**Insgesamt (n = 178)**		**p-Wert**
	**n**	**%**	**n**	**%**	**n**	**%**	**n**	**%**	
**14 Tage vor der Infektion**									< 0,001
Am Arbeitsplatz	80	96,4[a]	21	75,0[a]	48	82,8[a]	149	88,2[a]	0,003
Nicht am Arbeitsplatz	3	3,6[a]	9	32,1[a]	6	10,3[a]	18	10,7[a]	< 0,001
Urlaub	2	2,4[a]	4	14,3[a]	9	15,5[a]	15	8,9[a]	0,015
Insgesamt	83	98,8	28	87,5	58	93,5	169	94,9	
**Arbeitsbelastung vor der Infektion**									0,339
Erhöht	33	41,8[a]	7	26,9[a]	14	25,9[a]	54	34,0[a]	0,118
Vermindert	4	5,1[a]	1	3,8[a]	5	9,3[a]	10	6,3[a]	0,529
Unverändert	46	58,2[a]	18	69,2[a]	36	66,7[a]	100	62,9[a]	0,469
Insgesamt	79	94,0	26	81,3	54	87,1	159	89,3	
**Betriebliche Information und Aufklärung über SARS-CoV-2**									0,031
Ausreichend	45	56,3[a]	25	80,6[a]	38	69,1[a]	108	65,1[a]	0,040
Unzureichend	26	32,5[a]	5	16,1[a]	11	20,0[a]	42	25,3[a]	0,111
Auf weiterer Sprache gewünscht	5	6,3[a]	0	0,0[a]	0	0,0[a]	5	3,0[a]	0,063
Insgesamt	80	95,2	31	96,9	55	88,7	166	93,3	
**Infektionsschutz-maßnahmen**									0,351

Verpflichtender MNS[b] bei Kontakt mit Patient/-innen	57	70,4[a]	24	80,0[a]	41	68,3[a]	122	71,3[a]	0,500
Allgemeine Pflicht zur Nutzung von MNS[b]	46	56,8[a]	19	63,3[a]	37	61,7[a]	102	59,6[a]	0,761
Verpflichtende Anwendung von MNS[b] für Patient/-innen	27	33,3[a]	15	50,0[a]	23	38,3[a]	65	38,0[a]	0,275
Räumliche Unterteilung von Risikozonen	27	33,3[a]	9	30,0[a]	21	35,0[a]	57	33,3[a]	0,894
Isolierzimmer	28	34,6[a]	12	40,0[a]	19	31,7[a]	59	34,5[a]	0,735
Regelmäßiges Lüften	18	22,2[a]	20	66,7[a]	26	43,3[a]	64	37,4[a]	< 0,001
Regelmäßige Flächendesinfektion	40	49,4[a]	21	70,0[a]	44	73,3[a]	105	61,4[a]	0,009
Keine der genannten	8	9,9[a]	0	0,0[a]	4	6,7[a]	12	7,0[a]	0,198
Insgesamt	81	96,4	30	93,8	60	96,8	171	96,1	
**Wahrgenommene Sicherheit am Arbeitsplatz**									< 0,001
Ausreichend	26	38,2[a]	21	75,0[a]	36	67,9[a]	83	55,7[a]	< 0,001
Unzureichend	42	61,8[a]	7	25,0[a]	17	32,1[a]	66	44,3[a]	< 0,001
Insgesamt	68	81,0	28	87,5	53	85,5	149	83,7	

[a] berechnet auf Basis gültiger Antworten
[b] Mund-Nasen-Schutz

Zusatztabelle Z2: SARS-CoV-2-Infektion und Diagnose bei dem an der Querschnittserhebung teilnehmenden medizinischen Personal, Frankfurt/Main, März - August 2020 (n = 178)

	Ort des Infektionskontakts								
	**Arbeitsplatz (n = 84)**		**Privat (n = 32)**		**Unbekannt (n = 62)**		**Insgesamt (n = 178)**		**p-Wert**
	**n**	**%**	**n**	**%**	**n**	**%**	**n**	**%**	
**Anlass für PCR-Test**									0,015
Status als Kontaktperson	27	32,9[a]	13	43,3[a]	11	19,0[a]	51	30,0[a]	0,044
Symptomatisch	50	61,0[a]	18	60,0[a]	38	65,5[a]	106	62,4[a]	0,825
Keine Kontaktperson oder Symptome	3	3,7[a]	3	10,0[a]	6	10,3°	12	7,1[a]	0,247
Durch Arbeitsstelle veranlasst	33	40,2[a]	3	10,0[a]	9	15,5[a]	45	26,5[a]	< 0,001
Betriebliche Routine	6	7,3[a]	2	6,7[a]	6	10,3[a]	14	8,2[a]	0,767
Durch Gesundheitsamt	9	11,0[a]	3	10,0[a]	2	3,4[a]	14	8,2[a]	0,260
Insgesamt	82	97,6	30	93,8	58	93,5	170	95,5	
**Anwesenheit am Arbeitsplatz zwischen Test und Ergebnis**									0,042
Ja	14	17,5[a]	0	0,0[a]	7	11,9[a]	21	12,4[a]	0,042
Nein	66	82,5[a]	31	100,0[a]	52	88,1[a]	149	87,6[a]	0,042
Insgesamt	80	95,2	31	96,9	59	95,2	170	95,5	
**Status als Kontaktperson in den 14 Tagen vor der Diagnose**									0,026
Ja, durch Gesundheitsamt kontaktiert	31	38,8[a]	11	36,7[a]	10	17,2[a]	52	31,0[a]	0,020
Ja, durch Gesundheitsamt Quarantäne angeordnet	18	22,5[a]	9	30,0[a]	7	12,1[a]	34	20,2[a]	0,110
Keine Kontaktaufnahme durch Gesundheitsamt vor Diagnose	49	61,3[a]	19	63,3[a]	48	82,8[a]	116	69,0[a]	0,020
Insgesamt	80	95,2	30	93,8	58	93,5	168	94,4	
**Dauer der Erkrankung**									0,538

Weniger als eine Woche	8	9,8[a]	4	12,9[a]	10	16,9[a]	22	12,8[a]	0,451
Eine bis zwei Wochen	30	36,6[a]	15	48,4[a]	17	28,8[a]	62	36,0[a]	0,183
Zwei bis drei Wochen	23	28,0[a]	5	16,1[a]	11	18,6[a]	39	22,7[a]	0,265
Drei bis vier Wochen	10	12,2[a]	5	16,1[a]	8	13,6[a]	23	13,4[a]	0,859
Mehr als vier Wochen	8	9,8[a]	1	3,2[a]	9	15,3[a]	18	10,5[a]	0,200
Asymptomatisch	3	3,7[a]	1	3,2[a]	4	6,8[a]	8	4,7[a]	0,629
Insgesamt	82	97,6	31	96,9	59	95,2	172	96,6	
**Persistierende COVID-19-Symptome[b]**									0,108
Ja, mit Arbeitsunfähigkeit	1	1,3[a]	0	0,0[a]	1	1,8[a]	2	1,2[a]	0,774
Anhaltende Symptome im Zusammenhang mit COVID-19	20	25,6[a]	3	10,0[a]	14	24,6[a]	37	22,4[a]	0,194
Leichte Symptome mit verminderter Leistungsfähigkeit	21	26,9[a]	4	13,3[a]	6	10,5[a]	31	18,8[a]	0,038
Erhöhte psychische Belastung durch die Infektion	15	19,2[a]	4	13,3[a]	3	5,3[a]	22	13,3[a]	0,062
Keine persistierenden Symptome und volle Leistungsfähigkeit	43	55,1[a]	21	70,0[a]	38	66,7[a]	102	61,8[a]	0,235
Insgesamt	78	92,2	30	93,8	57	91,9	165	92,7	
**Transmission von SARS-CoV-2**									0,269
Potenziell eine andere Person infiziert	31	42,5[a]	8	26,7[a]	19	33,3[a]	58	36,3°	0,760
Potenziell Patient/-innen infiziert	7	9,6[a]	1	3,3[a]	3	5,3[a]	11	6,9[a]	0,436
Potenziell Kolleg/-innen infiziert	9	12,3[a]	1	3,3[a]	5	8,8[a]	15	9,4[a]	0,356
Potenziell Person im Haushalt infiziert	21	28,8[a]	6	20,0[a]	14	24,6[a]	41	25,6[a]	0,634

Potenziell Bekannte/-n infiziert	10	13,7[a]	4	13,3[a]	3	5,3[a]	17	10,6[a]	0,261
Keine Kenntnis, andere Person infiziert zu haben	42	57,5[a]	22	73,3[a]	38	66,7[a]	102	63,7[a]	0,269
Insgesamt	73	86,9	30	93,8	57	91,9	160	90,0	
**Faktoren die zu eigener Infektion beigetragen haben**									0,157
Annahme, sich nicht zu infizieren	11	16,4[a]	5	41,7[a]	9	29,0[a]	25	22,7[a]	0,097
Unregelmäßige Verwendung von PSA[c]	9	13,4[a]	0	0,0[a]	8	25,8[a]	17	15,5[a]	0,084
Kurzzeitiger Verzicht auf PSA[c] bei risikoreichem Kontakt	6	9,0[a]	0	0,0[a]	0	0,0[a]	6	5,5[a]	0,130
Geringe Verfügbarkeit von PSA[c]	29	43,3[a]	3	25,0[a]	10	32,3[a]	42	38,2[a]	0,353
Mangel an adäquater PSA[c]	37	55,2[a]	2	16,7[a]	14	45,2[a]	53	48,2[a]	0,045
Verwendung von defekter PSA[c]	3	4,5[a]	1	8,3[a]	1	3,2[a]	5	4,5[a]	0,770
Wiederverwendung von Einmal-PSA[c]	22	32,8[a]	0	0,0[a]	4	12,9[a]	26	23,6[a]	0,012
Unzureichende Schulung zur Anwendung der PSA[c]	11	16,4[a]	1	8,3[a]	5	16,1[a]	17	15,5[a]	0,769
Unzureichende Möglichkeiten für grundlegende Hygienemaßnahmen	5	7,5[a]	0	0,0[a]	2	6,5[a]	7	6,4[a]	0,621
Unzureichende Schulung in grundlegenden Hygienemaßnahmen	6	9,0[a]	0	0,0[a]	4	12,9[a]	10	9,1[a]	0,418
Unzureichende Umstrukturierung von Prozessen und Räumlichkeiten am Arbeitsplatz	27	40,3[a]	1	8,3[a]	7	22,6[a]	35	31,8[a]	0,039
Erhöhte arbeitsbedingte körperliche Erschöpfung	18	26,9[a]	4	33,3[a]	6	19,4[a]	28	25,5[a]	0,585

Erhöhte arbeitsbedingte psychische Erschöpfung	18	26,9[a]	4	33,3[a]	4	12,9[a]	26	23,6[a]	0,224
Erhöhte psychische Belastung durch die Pandemie	11	16,4[a]	2	16,7[a]	3	9,7[a]	16	14,5[a]	0,663
Unzureichende Arbeitsschutzmaßnahmen	26	38,8[a]	1	8,3[a]	9	29,0[a]	36	32,7[a]	0,102
Insgesamt	67	79,8	12	37,5	31	50,0	110	61,8	

[a] berechnet auf der Grundlage gültiger Antworten

[b] zum Zeitpunkt der Datenerhebung, die Infektionen lagen je nach Person ein bis sechs Monate zurück

[c] Persönliche Schutzausrüstung
